# Viral Regulation on Bacterial Community Impacted by Lysis-Lysogeny Switch: A Microcosm Experiment in Eutrophic Coastal Waters

**DOI:** 10.3389/fmicb.2019.01763

**Published:** 2019-07-31

**Authors:** Xiaowei Chen, Ruijie Ma, Yunlan Yang, Nianzhi Jiao, Rui Zhang

**Affiliations:** ^1^State Key Laboratory of Marine Environmental Science, College of Ocean and Earth Sciences, Institute of Marine Microbes and Ecospheres, Xiamen University, Xiamen, China; ^2^College of the Environment and Ecology, Xiamen University, Xiamen, China

**Keywords:** marine virus, viral life strategy, lysis, lysogeny, bacterial community

## Abstract

Marine viruses are major drivers of global biogeochemical cycles and energy fluxes, yet the importance of viral impacts on the succession and diversity of the bacterial community remains largely unexplored. Here, we explored viral life strategy and its potential effect on the bacterial community by experimental incubations of eutrophic coastal waters under lysogen-induced and non-induced treatments. The lysogen-induced treatment showed relatively constant viral and bacterial abundances, lytic and lysogenic viral production throughout the experimental period, together with the progressive declines in not only the relative abundances for SAR11, *Rhodobacteraceae*, *Alteromonadaceae*, and SAR86 but the bacterial community diversity. Conversely, the non-induced treatment observed the marked variation in the abundances of viruses, bacteria and cells with high nucleic acid content over the time course of incubation, which was congruent with the drastic shift in lytic and lysogenic viral production as well as the succession of bacterial community. Our results supported the hypotheses that a high level of lysogeny would occur with the increasing density of bacteria with rapid growth rate, which may contribute to a relatively lower host community diversity, whereas the lysogeny to lysis switching would fuel growth opportunities for less-active or initially rare bacterial taxa and generate a more diverse bacterial community. Altogether, the present study underscored the crucial regulatory role of the viral lysis-lysogeny pattern in bacterial community dynamics, composition and diversity, highlighting the viral impact on the microbial food web and biogeochemical processes.

## Introduction

Microbes are the keystone in marine ecosystem functions and play a central role in the microbial food web and elemental cycling in the ocean (Falkowski et al., 2008). Viruses manipulate microbe-driven biogeochemical processes by exerting a pivotal impact on microbial dynamics, metabolism, evolution, and community composition (Suttle, 2007; Zhang et al., 2007; Dell’Anno et al., 2015; Hurwitz and U’Ren, 2016; Cai et al., 2019). As important agents that transform the microbial biomass into dissolved organic matter by viral shunt (Wilhelm and Suttle, 1999), viruses greatly affect ocean carbon sequestration through biological and microbial carbon pumps (Suttle, 2005; Jiao et al., 2010; Laber et al., 2018). The impacts of viruses on the microbial community are mostly constrained by the patterns in viral life strategy (Weinbauer, 2004; Paul, 2008; Howard-Varona et al., 2017). Lytic viral infection is characterized by rapid production of new viral particles upon the lysis of the host, whereas lysogenic infection allows integration of temperate viruses into the host’s replicon (which remain as prophages within the bacteria called lysogens) that impacts host metabolism but can cause host lysis if the lytic cycle is triggered (Jiang and Paul, 1996; Salmond and Fineran, 2015).

Most previous studies of virus-host ecological interactions have focused on lytic viral infection and the subsequent impact of virus-mediated mortality on the microbial food web (Winget et al., 2011; Rastelli et al., 2016; Keshri et al., 2017; Liu H. et al., 2017; Wei et al., 2019). Generally, ecosystems with a higher bacterial abundance or activity would support a higher level of lytic viral production (lytic VP) (Rowe et al., 2008; Winget et al., 2011; De Corte et al., 2012) following the well-known density-dependent “Kill-the-Winner” model, indicating that viruses prefer to lyse the more dominant and rapidly growing microbes (Thingstad and Lignell, 1997; Winter et al., 2010). Conversely, a higher level of lysogenic viral production (lysogenic VP) usually appears with a lower bacterial abundance or activity in marine ecosystems, and lysogeny has been previously hypothesized to be a preferable survival strategy for both the virus and the host in harsh environments (Paul, 2008; Payet and Suttle, 2013; Brum et al., 2016; Chen et al., 2019). Recently, a newly proposed “Piggyback-the-Winner” model suggested that lysogeny would also be increasingly favored with a higher microbial density or activity (Knowles et al., 2016; Coutinho et al., 2017). This new insight into marine lysogeny contrasted with the traditional understanding of viral dynamics and their interactions with host microbes (Weinbauer, 2004; Paul, 2008; Winter et al., 2010), and revealed the intricate nature of marine lytic and lysogenic virus-host interactions. Thus, more research works is needed to unveil the roles of the viral lysis-lysogeny pattern in regulating the bacterial community.

High viral lysis pressure on the host has been confirmed to exert a major impact on bacterial diversity and the community structure through suppressing fast-growing bacteria, which are more vulnerable to viral attack (Malits and Weinbauer, 2009; Pradeep Ram and Sime-Ngando, 2008, 2010; Pradeep Ram et al., 2016; Liu H. et al., 2017). This process potentially provides growth opportunities for initially rare microbial populations and generally contributes to a more diverse host community (Thingstad and Lignell, 1997; Winter et al., 2010). However, the relative importance of lysogenic viral infection in influencing the bacterial community remains largely unclear (Weinbauer, 2004; Howard-Varona et al., 2017). There is a growing evidence that lysogeny may confer a better fitness in terms of competitiveness, virulence and resistance to environmental stress (Wang et al., 2010; Touchon et al., 2016; Ahmad et al., 2017; Lai et al., 2018). Consequently, lysogenic fitness, such as better growth efficiency, has been predicted to lead to a bacterial community dominated by lysogens, which are less affected by lytic virus attack through superinfection immunity (Weinbauer, 2004; Salmond and Fineran, 2015; Argov et al., 2017; Coutinho et al., 2017). This hypothesis suggests roles of lysogeny as a double-edged sword in regulating the bacterial community in which it not only functions as a “time bomb” through its lysogen-induced killing effect but also promotes a “win-win” strategy to help the host survive and compete (Paul, 2008; Howard-Varona et al., 2017; Breitbart et al., 2018).

Hence, to explore the patterns and relative importance of lytic and lysogenic virus-host interactions and their potential effects on functioning of the bacterial community, experimental incubations of natural coastal seawater under lysogen-induced and non-induced treatments were conducted in microcosms. The results will contribute to the exploration of the unresolved question of whether the viral lysis-lysogeny pattern triggers diversity changes and succession in marine bacterial community.

## Materials and Methods

### Experimental Set-Up

Seawater for the experiment was collected from a depth of approximately 1 m on April 20, 2016, from Xiamen Bay (24.5152°N, 118.2887°E; Supplementary Figure S1). Starting in 2012, we conducted long-term monthly observations at Xiamen Bay (Chen et al., 2019). The sampling site for this study was located at the eutrophic coastal region facing a lower riverine input and terrigenous pollution according to our previous investigations (Liu L. et al., 2017; Wang et al., 2019). The collected eutrophic coastal waters were characterized by a temperature of 18.80°C and salinity of 28.80 (determined by the YSI Professional Plus multiparameter meter, Yellow Springs, OH, United States). The nutrient concentrations (measured by the PowerMon Kolorimeter AA3 instrument; Bran-Luebbe, Charlotte, NC, United States) had values of 3.23 ± 0.12, 47.08 ± 5.29, 1.17 ± 0.02, and 51.82 ± 3.02 μmol L^-1^ for ${\mathrm{NO}}_{2}^{–}$, ${\mathrm{NO}}_{3}^{–}$, ${\mathrm{PO}}_{4}^{3–}$, and ${\mathrm{SiO}}_{3}^{2–}$, respectively, which were compatible with the values previously reported for Xiamen Bay (Liu L. et al., 2017; Chen et al., 2019; Wang et al., 2019).

A total of 40 L of coastal seawater was collected in polycarbonate carboys that were previously treated with 0.1 N HCl for at least 48 h and rinsed with the sampled seawater. The collected seawater was filtered through a 3 μm polycarbonate filter (Millipore, Billerica, MA, United States) to reduce the presence of zooplankton or protists and then pooled together in a larger acid-washed tank and distributed into four acid-washed 10 L polycarbonate bottles (Nalgene, United States) that served as the experimental microcosms. The experimental design consisted of a lysogen-induced treatment in which mitomycin C (final concentration of 1 μg L^-1^; Sigma-Aldrich, United States) was added to the replicated 10 L to induce the prophages into the lytic cycle; for the non-induced treatment, the other replicated microcosms were maintained without the mitomycin C addition. The microcosm incubation experiment was conducted in the dark for 8 days in a temperature-regulated room (set at 20 ± 1°C). During the incubation time course, subsamples were collected from each microcosm to monitor changes in the nutrient concentrations, viral and bacterial abundances, viral production and the bacterial community composition. Since previous studies have revealed that the induced prophages can retain lytic activity to bacterial community (James et al., 2012, 2014; Hammerl et al., 2016) or newly establish lysogeny via integrated into non-lysogenic bacteria (James et al., 2012; Hoai et al., 2016) and re-infect host bacteria to lysogenization (Williamson et al., 2001), both lytic VP and lysogenic VP were measured in not only non-induced treatment but lysogen-induced treatment during incubation to better determine the viral dynamics. The nutrient concentrations at each treatment were quite constant throughout the experiment (Supplementary Figure S3), indicating that bacterial growth and viral production were not nutrient-limited during the incubation. This result validated previous findings that microbial activities were less reliant on the nutrients in eutrophic coastal waters (Findlay et al., 1991; Iriarte et al., 2003).

### Viral and Bacterial Abundances

During the 8-days incubation, subsamples of 2 mL were collected with a high-frequency sampling during the first 48 h (every 3 h at first 6 h and then every 6 h) and almost daily afterward (every 12 h before 72 h of incubation and then every 24 h) from each microcosm to determine the viral and heterotrophic bacterial abundances by flow cytometry as previously described (Marie et al., 1999; Liang et al., 2014). The samples were fixed with glutaraldehyde to a final concentration of 0.5% at room temperature for 15 min in the dark. After flash freezing in liquid nitrogen, the samples were stored at –80°C for later analysis. Viruses were counted (Supplementary Figure S2A) using the Epics Altra II flow cytometer (Beckman Coulter, United States) with a blue laser emitting at 488 nm after staining with SYBR Green I (Molecular Probe, United States). Fluorescent beads (Molecular Probes) with a diameter of 1 μm were added as an internal standard. The heterotrophic bacterial abundance was also measured by flow cytometry (Accuri C6, Becton and Dickinson, United States) after staining with SYBR Green I. Additionally, the flow cytometric plots of bacteria allowed to distinguish two major subgroups consisting of cells with high nucleic acid (HNA) and low nucleic acid (LNA) contents (Gasol et al., 1999; Garcia et al., 2018). Hence, the abundances of the HNA and LNA cells were also counted during the analysis (Supplementary Figure S2B). All flow cytometric data analyses were performed with the FlowJo vX.0.7 software (Tree Star, United States).

### Lytic and Lysogenic Viral Production

Lytic VP and lysogenic VP were measured at days 0, 1, 2, 5, and 8 of the experiment using the viral reduction approach and prophage induction assay (Wilhelm et al., 2002; Paul and Weinbauer, 2010; Weinbauer et al., 2010). Briefly, an approximately 500 mL water sample from each microcosm was filtered using tangential flow filtration (TFF) with a 0.22 μm pore-size polyvinylidene difluoride cartridge (Labscale, Millipore, United States) to generate a 50 mL bacterial concentrate and ca. 400 mL filtrate. Virus-free water was obtained by filtering the 0.22 μm filtrate with a 30 kDa polysulfone cartridge (Millipore). The bacterial concentrate was diluted with virus-free water and then gently mixed, distributed into four 50 mL sterile tubes and incubated at *in situ* temperature in the dark. Two of the tubes were kept without any manipulations to measure lytic VP, whereas mitomycin C (final concentration of 1 μg mL^-1^) was added in the other two tubes as the inducing agent for the viral lytic cycle to measure lysogenic VP. Subsamples of 1 mL in replicate for bacterial and viral enumeration were taken at 3 h intervals during the 24 h incubation period and counted by flow cytometry.

The viral production rate was calculated using the online program VIPCAL^1^ (Luef et al., 2009). Briefly, the rate of viral accumulation in the control tubes (without mitomycin C addition) under a reduced natural viral abundance could represent lytic VP. Lysogenic VP was calculated as the difference between the viral increase in the mitomycin C-treated and the control tubes. Since a loss or increase could occur among a portion of the bacteria during the bacterial concentration process, lytic VP and lysogenic VP were multiplied by the bacterial correction factor to compare viral production rates from different microcosms and sampling dates. This factor was calculated by dividing the initial bacterial abundance from different sampling dates of the microcosms by the bacterial concentrations of the tubes at the beginning of the viral production measurements.

### DNA Extraction, Sequencing and Analysis of the Bacterial Community

The bacterial community DNA used for the molecular analysis was obtained after 0, 1, 2, 5, and 8 days of incubation. Approximately 500 mL of seawater from each microcosm was collected onto 0.2 μm pore-size polycarbonate filters (47 mm, Millipore, United States) by vacuum filtration, flash frozen in liquid nitrogen and stored at -80°C. DNA was extracted using the PowerSoil DNA Isolation Kit (MoBio Laboratories, United States) following the manufacturer’s protocol. The V3-V4 hypervariable region of the bacterial 16S rRNA gene was used to evaluate the bacterial community composition by high-throughput sequencing with the primer pair 341F (5′-CCTAYGG GRBGCASCAG-3′) and 806R (5′-GGACTACNNGGGTATC TAAT-3′) complemented with sample-specific barcodes. The amplicons were sequenced with an Illumina MiSeq (Illumina, San Diego, CA, United States). Sequences that contained more than one ambiguous nucleotide that did not have a complete barcode and primer at one end or that were shorter than 200 bp after removal of the barcode and primer sequences were eliminated. Chimeric sequences were also identified and removed. After the quality filtering, denoising and removal of potential chimeras, a total of 724,533 high-quality sequences were retrieved. These paired-end sequences were clustered into operational taxonomic units (OTUs) based on an identity cutoff of 97% similarity and the taxonomy was assigned in Mothur (v.1.36.1) using the Silva 119 reference database (Schloss et al., 2009; Quast et al., 2013). The alpha diversity indices, including richness and the Shannon diversity index, were calculated from the OTUs. All sequencing data are available at the NCBI Sequence Read Archive (SRR8932524 to SRR8932543) under BioProject PRJNA533816.

### Statistical Analysis

The Shapiro–Wilk *W* test for data normality was applied prior to the analysis, and the data were log-transformed to meet normality when necessary. One-way analysis of variance (ANOVA) was performed with the SPSS 24.0 software (SPSS Inc., United States) to test significant differences of the lytic VP, lysogenic VP, bacterial community richness and Shannon diversity index among the different sampling dates. If significant differences (*P* < 0.05) were observed, the *post hoc* Tukey’s test was also performed. The Bray–Curtis similarities between different bacterial communities were calculated using PRIMER 6 (Primer-E, Plymouth, United Kingdom). Permutational analysis of multivariate variance (PERMANOVA) with 9999 permutations was also conducted by PRIMER 6 to assess significant difference among bacterial communities from different sampling dates. The Linear regression model was used in GraphPad Prism 7 (GraphPad, CA, United States) to characterize the following information (i) the relationships among viral, bacterial, HNA and LNA cell abundances; (ii) the relationships between viral production (lytic VP and lysogenic VP) and the host abundances (bacteria, HNA, and LNA cells).

## Results

### Viral and Bacterial Abundances

**FIGURE 1:**
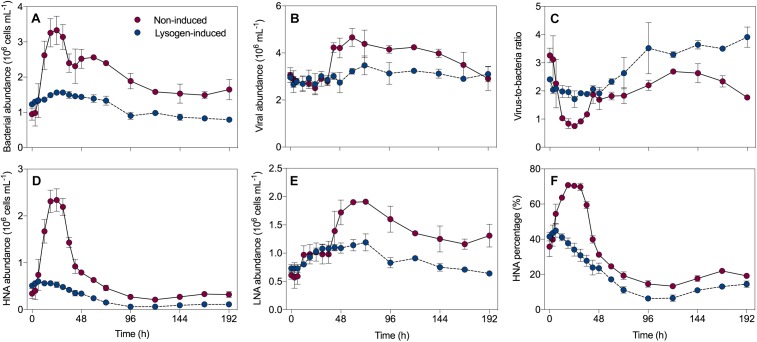
Dynamics overtime of the bacterial abundance **(A)**, viral abundance **(B)**, virus-to-bacteria ratio **(C)**, high nucleic acid (HNA) cells **(D)**, low nucleic acid (LNA) cells **(E)** and the percentage of HNA cells **(F)** in the non-induced and lysogen-induced treatments. The error bars are indicated as standard deviation (SD).

In the non-induced treatment, the bacterial abundance exhibited net growth during the first 24 h, with an increase from 0.95 ± 0.17 × 10^6^ cells mL^-1^ at the beginning to 3.33 ± 0.40 × 10^6^ cells mL^-1^ (Figure 1A), followed by a sharp decrease that reached 2.40 ± 0.26 × 10^6^ cells mL^-1^ at 36 h. After a slight recovery before 60 h, a continuous decline of the bacterial abundance was recorded. The viral abundance did not follow this pattern, with an initial value of 3.06 ± 0.31 × 10^6^ mL^-1^ that remained relatively stable to 36 h (Figure 1B). Thereafter, an abrupt 1.5-fold increase in the viral abundance from 2.79 ± 0.18 × 10^6^ mL^-1^ to 4.23 ± 0.20 × 10^6^ mL^-1^ was observed at 48 h, and then viruses continued to grow and reach a peak at 60 h. Consequently, this dynamic pattern of bacteria and viruses leaded to the virus-to-bacteria ratio showed a drastic decrease before 36 h, followed by a continuous ascent during the incubation in the non-induced treatment (Figure 1C). However, in the lysogen-induced treatment, the bacterial abundance varied slightly during the time course of incubation (Figure 1A). Similarly, the viral abundance and virus-to-bacteria ratio also did not show much variation over the whole duration of the experiment (Figure 1B,C).

The total bacteria were divided into two subgroups (HNA and LNA cells) using flow cytometry analysis. The HNA cells presumably represent a more active fraction of the community than the LNA cells (Gasol et al., 1999; Vila-Costa et al., 2012). Generally, the dynamics in the abundance of HNA cells mirrored that of the total bacterial abundance (Figure 1D), with a sharp increase from 0.34 ± 0.11 × 10^6^ cells mL^-1^ (0 h) to 2.34 ± 0.24 × 10^6^ cells mL^-1^ (24 h) and then a dramatic decline over the course of the incubation in the non-induced treatment, along with an overall decline in the lysogen-induced treatment. The abundance of LNA cells in the non-induced treatment, which had an initial value of 0.61 ± 0.07 × 10^6^ cells mL^-1^, stayed relatively stable during the first 36 h and showed net growth to reach a peak at approximately 60 h (Figure 1E). Ranging between 0.64 ± 0.02 × 10^6^ and 1.19 ± 0.15 × 10^6^ cells mL^-1^, the abundance of LNA cells in the lysogen-induced treatment was more stable. Consequently, the percentage of HNA cells within the total bacteria followed the dynamics of the total and HNA bacterial abundances (Figure 1F), with the contribution in the non-induced treatment reaching a peak as high as 70.75% at approximately 24 h and an overall decrease in the lysogen-induced treatment. Although the viral abundance over the course of the experiment showed no apparent correlation with the total bacterial abundance (Figure 6A), the dynamics of the viral abundance for each treatment was significantly negatively associated with the HNA abundance (Figure 6B and Supplementary Table S1).

### Lytic and Lysogenic Viral Production

**FIGURE 2:**
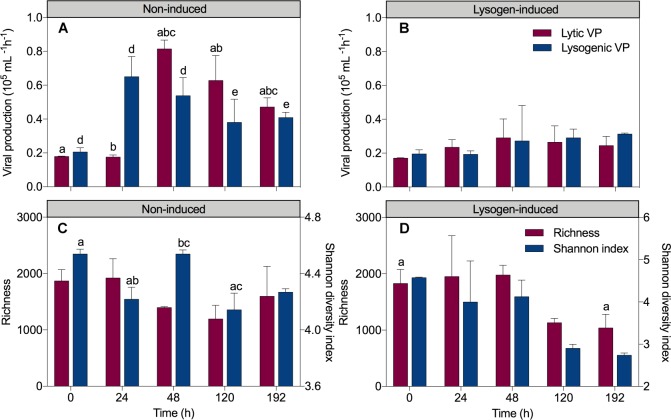
Dynamics overtime of lytic and lysogenic viral production in the non-induced treatment **(A)** and the lysogen-induced treatment **(B)** and the richness and Shannon diversity index in the non-induced treatment **(C)** and the lysogen-induced treatment **(D)**. The error bars are indicated as standard deviation (SD). One-way analysis of variance (ANOVA) with Turkey’s HSD *post hoc* test was used to compare the viral production, richness and Shannon diversity index at the different sampling time points. Samples with the same letters (a, b, c, d, and e) denote values that are significantly different (*P* < 0.05).

In the non-induced treatment, the sharp increase in the total bacteria and HNA abundances but invariant viral abundance after a short-term 24 h incubation coincided with a 3.2-fold increase in lysogenic VP (from 0.21 ± 0.03 × 10^5^ mL^-1^ h^-1^ at 0 h to 0.65 ± 0.12 × 10^5^ mL^-1^ h^-1^ at 24 h; *P* = 0.008; ANOVA) and unchanged lytic VP (0.18 ± 0.003 × 10^5^ mL^-1^ h^-1^ at 0 h to 0.17 ± 0.01 × 10^5^ mL^-1^ h^-1^ at 24 h) (Figure 2A). Conversely, a significant 4.7-fold increase in lytic VP (reached 0.82 ± 0.05 × 10^5^ mL^-1^h^-1^; *P* = 0.001, ANOVA) but a reduction in lysogenic VP (reached 0.54 ± 0.11 × 10^5^ mL^-1^h^-1^) was detected at 48 h, corresponding to a substantial increase in the viral abundance but a drop in the total bacteria and HNA abundances. Afterward, viral production suffered a slight continuous decline throughout the experiment, reaching 0.47 ± 0.05 × 10^5^ mL^-1^h^-1^ for lytic VP and 0.41 ± 0.03 × 10^5^ mL^-1^h^-1^ for lysogenic VP at 192 h. In the lysogen-induced treatment (Figure 2B), lytic VP varied slightly throughout the experimental period (between 0.17 ± 0.003 × 10^5^ mL^-1^h^-1^ and 0.29 ± 0.11 × 10^5^ mL^-1^h^-1^). With an initial value of 0.20 ± 0.02 × 10^5^ mL^-1^h^-1^, lysogenic VP also remained relatively constant during the whole time course of incubation (between 0.19 ± 0.02 × 10^5^ mL^-1^h^-1^ and 0.31 ± 0.01 × 10^5^ mL^-1^h^-1^).

The total bacterial abundance was significantly positively correlated with lysogenic VP in the non-induced treatment (*R*^2^ = 0.991, *P* < 0.001; linear regression), showing that more lysogeny occurred in the presence of more bacteria (Figure 6D and Supplementary Table S1). Similarly, a trend for a positive correlation was found between the HNA cells and lysogenic VP in the non-induced treatment (Figure 6E, *R*^2^ = 0.686, *P* = 0.083; linear regression), whereas the abundance of LNA cells was significantly positively correlated with lytic VP (Figure 6F, *R*^2^ = 0.794, *P* = 0.042; linear regression). No significant correlation was recorded between viral production and the total bacterial, HNA and LNA cells abundances in the lysogen-induced treatment.

### Bacterial Community Composition

**FIGURE 3:**
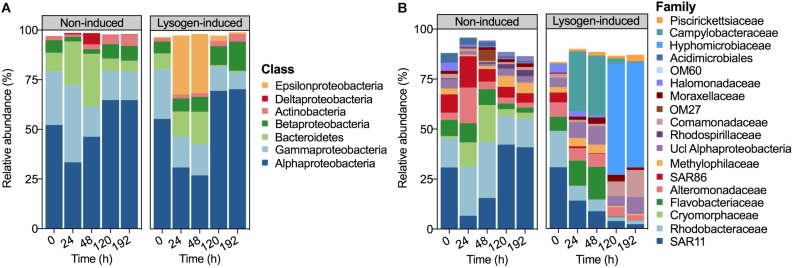
Bacterial community composition derived from 16S rRNA gene sequencing data demonstrating the relative abundances of the bacterial taxa at the class level **(A)** and family level **(B)** during incubation. Taxa comprising >1% of the total number of sequences are shown.

The bacterial community composition at the beginning of the experiment was dominated by Alphaproteobacteria (52.16%), followed by Gammaproteobacteria (27.11%), Bacteroidetes (9.31%) and Betaproteobacteria (6.32%) (Figure 3A). After 24 h of incubation, the relative abundances of Alphaproteobacteria (33.44%) and Betaproteobacteria (2.45%) declined in the non-induced treatment, whereas those of Gammaproteobacteria and Bacteroidetes increased to 38.97 and 21.81%, respectively. With the increasing incubation time, the relative abundances of Alphaproteobacteria and Betaproteobacteria recovered in the non-induced treatment, whereas Gammaproteobacteria and Bacteroidetes showed a continuous decrease. In contrast, the relative abundance of Epsilonproteobacteria in the lysogen-induced treatment was compatible with that of the dominant Alphaproteobacteria after 24 h (29.78%) and 48 h (29.75%) of incubation, whereas a sharp decline to less than 3% was noted at 120 h and 192 h of incubation.

At the family level, a larger shift in the bacterial community composition than at the class level was observed over the incubation (Figure 3B). The relative abundances of *Cryomorphaceae*, *Alteromonadaceae*, SAR86, and *Rhodobacteraceae* dominated the community (each population >10%) at 24 h in the non-induced treatment and showed substantial increases with 11.0, 5.0, 1.70 and 1.69-fold changes, respectively, relative to the beginning and to a greater extent than that of the initially dominant SAR11. However, the non-induced treatment showed a lasting drop in the relative abundances of *Alteromonadaceae*, *Cryomorphaceae*, *Rhodobacteraceae*, *Flavobacteriaceae*, and SAR86 and a continuous increase in SAR11 at the duration of incubation after 24 h of incubation, leading to a bacterial community structure similar to the initial status at the end of incubation (192 h). The relative abundances of SAR11, *Rhodobacteraceae*, *Alteromonadaceae*, and SAR86 showed continuous declines in the lysogen-induced treatment, whereas the *Flavobacteriaceae* and *Campylobacteraceae* were more abundant after 24 and 48 h of incubation, which was in contrast to the bacterial community dominated by *Hyphomicrobiaceae* after 120 and 192 h of incubation.

### Bacterial Community Diversity and Succession

**FIGURE 4:**
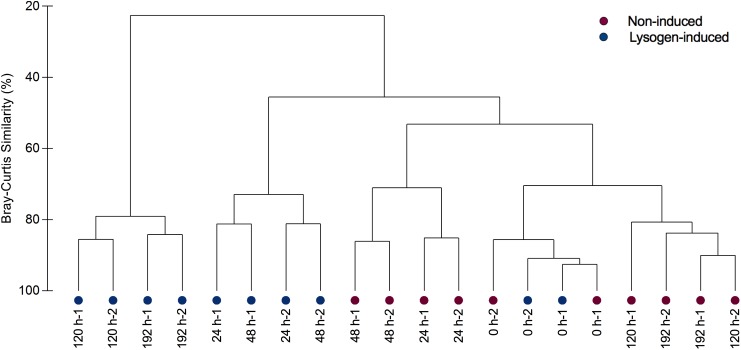
Clustering of bacterial communities from replicate samples over the course of incubation in the non-induced and lysogen-induced treatments based on Bray–Curtis similarities.

Analysis of the alpha-diversity metrics (richness and the Shannon index) also indicated a lasting transition in bacterial diversity during the experiment in the lysogen-induced treatment, with a progressive decline in the diversity index over time (Figure 2D). In contrast, a substantially different shift in the Shannon index was observed in the non-induced treatment (Figure 2C), with a significant drop at 24 h (from an initial value of 4.54 ± 0.03 to 4.22 ± 0.09; *P* = 0.03, ANOVA) that was in contrast to the significant increase from 24 to 48 h (4.54 ± 0.03; *P* = 0.03, ANOVA). To estimate beta-diversity among the bacterial communities, the cluster analysis was performed based on Bray–Curtis similarities (Figure 4). Bacterial communities after 24 and 48 h of incubation in the non-induced treatment showed the significant difference with the initial status (*P* = 0.025 for samples at 24 h and *P* = 0.03 for samples at 48 h, PERMANOVA), while both samples after 120 h and 192 h of incubation were more similar to the initial bacterial community (*P* > 0.05, PERMANOVA). In the lysogen-induced treatment, conversely, the bacterial communities collected at 24 h was more similar to the initial sample (*P* > 0.05, PERMANOVA) than to those significantly different bacterial communities collected after a longer incubation (*P* = 0.01, 0.004, and 0.006 for samples at 48, 120, and 192 h, respectively, PERMANOVA).

**FIGURE 5:**
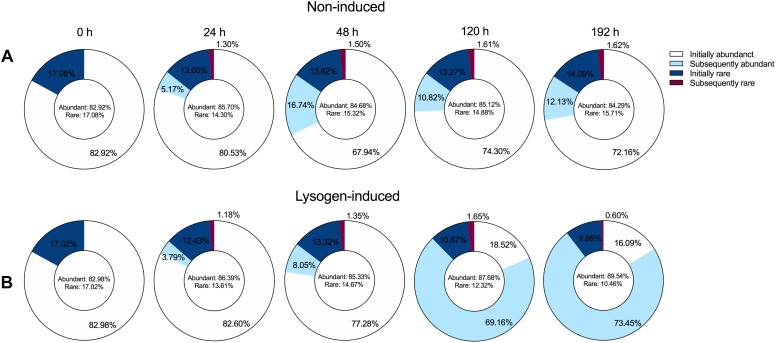
Dynamics overtime of the proportion of the abundant (relative abundance ≥0.1%) and rare OTUs (relative abundance <0.1%) among the overall OTUs pools in the non-induced treatment **(A)** and lysogen-induced treatment **(B)**. The initially abundant and rare OTUs represent the abundant and rare OTUs at the beginning of the experiment (0 h). Among the total abundant OTUs, the subsequently abundant OTUs indicate that these abundant OTUs are not initially abundant at 0 h but are newly generated during incubation. Among the total rare OTUs, the subsequently rare OTUs indicate that these rare OTUs are initially abundant at 0 h but become rare OTUs after incubation.

To explore the dynamic pattern of the abundant and rare bacterial members among the community during the incubation, we inspected the proportions of bacterial taxa that were initially abundant and rare OTUs or that subsequently became abundant and rare OTUs among the OTUs pools (Figure 5), following the abundance threshold definition from the previous study (Shen et al., 2018). The bacterial community at the beginning of the experiment harbored ca. 83% abundant and ca. 17% rare OTUs. Despite the large shift in the bacterial community composition (Figure 3), the booming bacterial and HNA abundances (Figure 1) and the markedly increased lysogenic VP (Figure 2) after 24 h of incubation in the non-induced treatment, only 5.17% of the initial rare OTUs at 0 h changed their statuses and became abundant at 24 h (Figure 5A). However, the proportion of subsequently abundant OTUs largely increased and retained a certain quota during the subsequent incubation in the non-induced treatment, reaching 16.74, 10.82, and 12.13% at 48, 120, and 192 h, respectively. In the lysogen-induced treatment, the proportion of subsequent abundant OTUs increased over time (Figure 5B), especially at 120 and 192 h when the *Hyphomicrobiaceae* and *Comamonadaceae* populations dominated the bacterial community.

## Discussion

### Lysogenic Fitness Helps With Competition

In the non-induced treatment, the bacterial abundance after 24 h of incubation showed a sudden bloom that was mostly contributed by the HNA cells with a high proportion of HNA cells reaching 70.75% (Figure 1). Flow cytometric sorting have well characterized the HNA fraction, which was mostly composed of the more active members with high growth rates and activity, such as *Rhodobacterales* and Bacteroidetes groups. Conversely, the LNA cells were primarily represented by members predicted to have low adaptability and replication rates, such as SAR11 (Gasol et al., 1999; Schattenhofer et al., 2011; Vila-Costa et al., 2012; Baltar et al., 2016; Pradeep Ram et al., 2016). The fast-growing populations, especially taxa belonging to *Alteromonadaceae* and *Rhodobacteraceae*, which are major players in the drawdown of labile carbon in the ocean (Zheng et al., 2018), have been found to rapidly proliferate in response to environmental changes (Buchan et al., 2014). After 24 h of incubation, the strong increase in bacterial abundance and HNA cells together with the booming and dominance of rapidly growing populations, such as *Alteromonadaceae*, *Cryomorphaceae*, and *Rhodobacteraceae*, over the less-active SAR11 clade confirmed that the HNA cells were more active members and suggested that this subgroup was the winner in competing for resources (Figure 1, 3; Eilers et al., 2000; Suttle, 2007; Xu et al., 2013; Kirchman, 2016).

Although generally the metabolically active members of bacterial communities are considered as the preferable hosts for selective lytic viral infection to produce more viral progeny (Weinbauer, 2004; Suttle, 2005), the marked increase in active bacteria after short-term incubation did not stimulate lytic VP but instead significantly enhanced the lysogeny (Figure 2A). These results suggested that most of the appearing dominant fast-growing bacteria potentially belonged to lysogens, because lysogenic fitness might help lysogens compete and hence dominate the community. Previous study has revealed that bacteria with higher growth rates contained a higher frequency of prophages and were characterized as lysogens (Touchon et al., 2016). Additionally, compared to those of the non-lysogenic cells, lysogens are well documented to confer more fitness in terms of competitiveness, substrate utilization, biofilm formation, glycogen accumulation and resistance to environmental stress (Wang et al., 2010; Ahmad et al., 2017; Lai et al., 2018). In addition to the ability of prophages to change the phenotypic properties of their hosts, establishment of lysogeny also confers immunity against lytic attack by the same or close viruses, facilitating domination of the bacterial community by lysogeny (Weinbauer, 2004; Salmond and Fineran, 2015; Argov et al., 2017). These effects result in less lysis pressure through lysogenic superinfection immunity and a reduction in reproduction of viral progeny (Weinbauer, 2004; Paul, 2008; Bondy-Denomy and Davidson, 2014), leading to an invariant viral abundance and lytic VP during the bacterial bloom (Figure 1, 2).

Here, our results indicated that the benefits of lysogenic fitness also existed in natural bacterial assemblages, probably by helping lysogens confer selective advantages that would eventually become abundant. Viruses can “domesticate” their host microbes through lysogeny instead of killing them, resulting in a mutualistic “win-win” relationship rather than an antagonistic “arm race” for both of them, protecting the hosts from new viral infections by other viral populations (Breitbart, 2012; Thingstad et al., 2014). Hence, the higher abundance of bacteria with higher fraction of active members was coupled with more lysogenic infection and a reduction in the viral density (Figure 1–3). This finding was in contrast to the previously determined negative relationship between bacterial activities and the frequency of lysogenic cells found in natural or experimental conditions, especially in harsh marine environments (Jiang and Paul, 1996; Weinbauer, 2004; Paul, 2008; Payet and Suttle, 2013; Chen et al., 2019). Conversely, this finding agreed with the “Piggyback-the-Winner” model where high lysogeny could appear with a high abundance of the bacterial host that potentially has a higher growth rate (Knowles et al., 2016; Coutinho et al., 2017).

### Lysogeny to Lysis Switching Fueled the Growth of Less Active Bacteria

Viral lysis has been confirmed as one dominant top-down control that regulates bacterial dynamics, potentially contributing to the mortality of up to 50% of the new bacteria produced daily (Suttle, 2005). Lysogeny can affect the bacterial community by expanding the distribution of lysogens via their acquired lysogenic fitness, however, the existence of lysogeny in the ecosystem may also serve as a “time bomb,” since the prophage will strongly shape the bacterial community structure by re-entering the lytic cycle when induced (Hewson and Fuhrman, 2007; Paul, 2008). The sharp drop in bacterial abundance (Figure 1), the proportion of HNA cells, the relative abundance of rapidly growing populations (Figure 3), such as *Alteromonadaceae* and *Rhodobacteraceae*, and the substantial increase in viral abundance after 36 h of incubation in the non-induced treatment together with the markedly enhanced lytic VP but reduced lysogenic VP (Figure 2), indicating the appearance of lysogeny to lysis switching that caused the major lysis event in the system.

**FIGURE 6:**
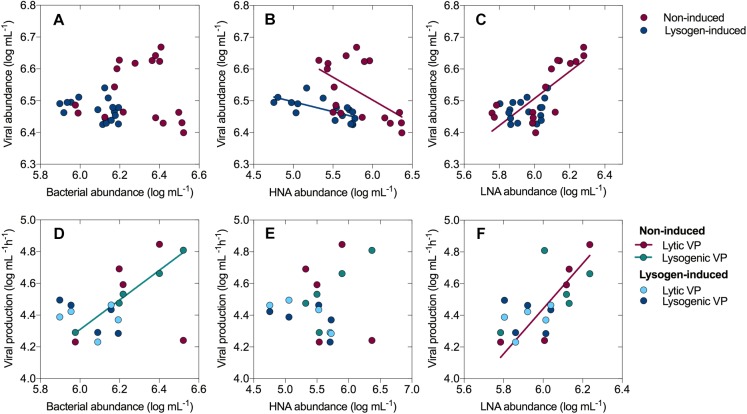
Relationships between viral abundance and bacterial abundance **(A)**, the abundances of HNA cells **(B)** and LNA cells **(C)**. Relationships between viral production (lytic and lysogenic) and bacterial abundance **(D)**, the abundances of HNA cells **(E)** and LNA cells **(F)**. Only the linear regression with a significance level of *P* < 0.05 are shown in the graph.

The lysogeny to lysis switching was coupled with a shift in dominance from HNA to LNA cells and the continuous decline in the fractions of active members, such as *Alteromonadaceae*, but accumulation of slow-growing populations, such as SAR11 clade (Figure 1–3), leading to a situation in which more lytic VP would stimulate more LNA cells (Figure 6). These results suggested that selective removal of metabolically active bacterial assemblages through viral lysis could benefit the less active fraction, perhaps via the use of lysate for its growth and survival and reducing competition among the community (Xu et al., 2013; Sheik et al., 2014; Pradeep Ram et al., 2016). The highly inducible prophages caused massive cell lysis and the potential release of labile dissolved organic matter, thereby enhancing the utilization of this lysate by living bacteria and fueling heterotrophic recycling of organic carbon and nutrient regeneration within the microbial loop (Azam et al., 1983; Wilhelm and Suttle, 1999; Jiao et al., 2010). Consequently, the lysogeny to lysis switching triggered a growth opportunity for the originally rare bacterial taxa (Figure 5), suggesting that rare bacteria can become dominant when previously dominant bacterial are killed by viruses, following the “Kill-the-Winner” regulation hypothesis (Thingstad and Lignell, 1997; Winter et al., 2010).

### Effects of the Viral Lysis-Lysogeny Pattern on the Bacterial Community

Heterogeneous mortality mediated by viral lysis is well reported to be crucial for both shaping the microbial community composition and diversifying bacterial species (Thingstad and Lignell, 1997; Weinbauer, 2004; Suttle, 2007). Previous field investigations and model studies on the influence of viral infection on dynamics in bacterial community composition and diversity have mostly focused on the lytic property and are mainly based on the assumption that viral lysis regulates the bacterial community structure as predicted by the “Kill-the-Winner” hypothesis (Thingstad and Lignell, 1997). According to this hypothesis, the fastest growing bacterial population is the most susceptible to viral-mediated mortality, and the viral lysis facilitates the release of cellular labile carbon resources that becomes available for less competitive species; in this way, bacterial diversity is promoted and community composition is changed (Winter et al., 2010; Thingstad et al., 2014). In contrast, the “Piggyback-the-Winner” hypothesis is based more on viral lysogenic infection providing another scenario in which a high level of lysogeny may reduce the bacterial diversity and also lead to the bacterial community succession, since lysogens that resist superinfection by relative viruses will become increasingly important and abundant among the bacterial community (Knowles et al., 2016). The significantly different bacterial community compositions were found tightly related to the viral lysis-lysogeny switch in the non-induced treatment (Figure 2–4), confirming the pivotal impact on bacterial community exerted by the dynamic pattern in both lytic and lysogenic viral infection. Compared to non-induced treatment, the induction of lysogens did substantially affect the bacterial growth and community composition, suggesting the significant impact of lysogeny on bacterial community. Such alteration might also be partially explained by the potential effect of inducing reagent itself on bacterial community since mitomycin C might inhibit bacterial growth and certain bacterial clades would be stimulated by undetermined compounds in the mitomycin C (Hewson and Fuhrman, 2007).

Our results observed the coupling of the viral lysis-lysogeny pattern and bacterial diversity, showing a high level of lysogeny accorded with dominance of active bacterial groups among the community with a lower bacterial Shannon index; in contrast, a higher bacterial Shannon index was found during the period with more viral lysis (Figure 2). The lysogen-dominant bacterial community would suffer a significant reduction in bacterial diversity, whereas the subsequent viral lysogeny to lysis switching due to prophage-induced direct killing of host bacteria would contribute to a dramatic increase in bacterial community diversity (Knowles et al., 2016; Pradeep Ram et al., 2016; Liu H. et al., 2017; Rastelli et al., 2017). During the experimental incubation, the succession of the bacterial community toward the initial status was observed after the lysogeny to lysis switching (Figure 3, 4), suggesting the importance of viruses in sustaining the bacterial community. Hence, our results strongly indicated that the “Kill-the-Winner” and “Piggyback-the-Winner” dynamic patterns occurred jointly rather than as mutually exclusive viral control strategies and thereby shaped the succession and dynamics of the bacterial community.

Taken together, this study contributed to understanding of the impact of the viral life strategy on the bacterial community, indicating that the benefits of lysogenic fitness probably helped lysogens confer selective advantages for competition and dominance in the community. Additionally, our findings noted that the influence of viral lysis-lysogeny switch on the bacterial community dynamics, composition and diversity could be more important than previously thought. Future investigations should focus on deepening information on the multiple factors affecting the viral lysis-lysogeny patterns and exploring the roles of lysogenic virus-host interactions in the functioning of the marine microbial communities and biogeochemical processes.

## Data Availability

Publicly available datasets were analyzed in this study. This data can be found here: https://www.ncbi.nlm.nih.gov/bioproject/PRJNA533816.

## Author Contributions

RZ and XC designed and coordinated the study, and analyzed the results. XC, RM, and YY performed the experiments and data analyses. XC and RZ wrote the manuscript. All authors interpreted the data and suggested the improvements on the manuscript.

## Conflict of Interest Statement

The authors declare that the research was conducted in the absence of any commercial or financial relationships that could be construed as a potential conflict of interest.

## Footnotes

1 http://www.univie.ac.at/nuhag-php/vipcal/

## References

[B1] Ahmad A. A. Stulberg M. J. Huang Q. (2017). Prophage Rs551 and its repressor gene orf14 reduce virulence and increase competitive fitness of its ralstonia solanacearum carrier strain UW551. *Front. Microbiol.* 8:2480. 10.3389/fmicb.2017.02480 29312189 PMC5744446

[B2] Argov T. Azulay G. Pasechnek A. Stadnyuk O. Ran-Sapir S. Borovok I. (2017). Temperate bacteriophages as regulators of host behavior. *Curr. Opin. Microbiol.* 38 81–87. 10.1016/j.mib.2017.05.002 28544996

[B3] Azam F. Fenchel T. Field J. G. Gray J. S. Meyer-Reil L. A. Thingstad F. (1983). The ecological role of water-column microbes in the sea. *Mar. Ecol. Prog. Ser.* 10 257–263. 10.3354/meps010257

[B4] Baltar F. Palovaara J. Unrein F. Catala P. Horňák K. Šimek K. (2016). Marine bacterial community structure resilience to changes in protist predation under phytoplankton bloom conditions. *ISME J.* 10 568–581. 10.1038/ismej.2015.135 26262814 PMC4817682

[B5] Bondy-Denomy J. Davidson A. R. (2014). When a virus is not a parasite: the beneficial effects of prophages on bacterial fitness. *J. Microbiol.* 52 235–242. 10.1007/s12275-014-4083-3 24585054

[B6] Breitbart M. (2012). Marine viruses: truth or dare. *Annu. Rev. Mar. Sci.* 4 425–448. 10.1146/annurev-marine-120709-142805 22457982

[B7] Breitbart M. Bonnain C. Malki K. Sawaya N. A. (2018). Phage puppet masters of the marine microbial realm. *Nat. Microbiol.* 3 754–766. 10.1038/s41564-018-0166-y 29867096

[B8] Brum J. R. Hurwitz B. L. Schofield O. Ducklow H. W. Sullivan M. B. (2016). Seasonal time bombs: dominant temperate viruses affect Southern Ocean microbial dynamics. *ISME J.* 10 437–449. 10.1038/ismej.2015.125 26296067 PMC4737935

[B9] Buchan A. LeCleir G. R. Gulvik C. A. González J. M. (2014). Master recyclers: features and functions of bacteria associated with phytoplankton blooms. *Nat. Rev. Micro.* 12 686–698. 10.1038/nrmicro3326 25134618

[B10] Cai L. Jørgensen B. B. Suttle C. A. He M. Cragg B. A. Jiao N. (2019). Active and diverse viruses persist in the deep sub-seafloor sediments over thousands of years. *ISME J.* 433:861. 10.1038/s41396-019-0397-9 30877284 PMC6776017

[B11] Chen X. Wei W. Wang J. Li H. Sun J. Ma R. (2019). Tide driven microbial dynamics through virus-host interactions in the estuarine ecosystem. *Water Res.* 160 118–129. 10.1016/j.watres.2019.05.051 31136846

[B12] Coutinho F. H. Silveira C. B. Gregoracci G. B. Thompson C. C. Edwards R. A. Brussaard C. P. D. (2017). Marine viruses discovered via metagenomics shed light on viral strategies throughout the oceans. *Nat. Commun.* 8:15955. 10.1038/ncomms15955 28677677 PMC5504273

[B13] De Corte D. Sintes E. Yokokawa T. Reinthaler T. Herndl G. J. (2012). Links between viruses and prokaryotes throughout the water column along a North Atlantic latitudinal transect. *ISME J.* 6 1566–1577. 10.1038/ismej.2011.214 22258100 PMC3400414

[B14] Dell’Anno A. Corinaldesi C. Danovaro R. (2015). Virus decomposition provides an important contribution to benthic deep-sea ecosystem functioning. *Proc. Natl. Acad. Sci. U. S. A.* 112 E2014–E2019. 10.1073/pnas.1422234112 25848024 PMC4413343

[B15] Eilers H. Pernthaler J. Amann R. (2000). Succession of pelagic marine bacteria during enrichment: a close look at cultivation-induced shifts. *Appl. Environ. Microbiol.* 66 4634–4640. 11055904 10.1128/aem.66.11.4634-4640.2000 PMC92360

[B16] Falkowski P. G. Fenchel T. DeLong E. F. (2008). The Microbial Engines That Drive Earth’s Biogeochemical Cycles. *Science* 320 1034–1039. 10.1126/science.1153213 18497287

[B17] Findlay S. Pace M. L. Lints D. Cole J. J. Caraco N. F. Peierls B. (1991). Weak coupling of bacterial and algal production in a heterotrophic ecosystem: the Hudson River estuary. *Limnol. Oceanogr.* 36 268–278. 10.4319/lo.1991.36.2.0268

[B18] Garcia F. C. Calleja M. L. Al-Otaibi N. Røstad A. Morán X. A. G. (2018). Diel dynamics and coupling of heterotrophic prokaryotes and dissolved organic matter in epipelagic and mesopelagic waters of the central Red Sea. *Environ. Microbiol.* 20 2990–3000. 10.1111/1462-2920.14336 30051643

[B19] Gasol J. M. Zweifel U. L. Peters F. Fuhrman J. A. Hagstrom A. (1999). Significance of size and nucleic acid content heterogeneity as measured by flow cytometry in natural planktonic bacteria. *Appl. Environ. Microbiol.* 65 4475–4483. 10508078 10.1128/aem.65.10.4475-4483.1999 PMC91596

[B20] Hammerl J. A. Göllner C. Dahouk Al S. Nöckler K. Reetz J. Hertwig S. (2016). Analysis of the First Temperate Broad Host Range Brucellaphage (BiPBO1) Isolated from B. inopinata. *Front. Microbiol.* 7:24. 10.3389/fmicb.2016.00024 26858702 PMC4729917

[B21] Hewson I. Fuhrman J. A. (2007). Characterization of lysogens in bacterioplankton assemblages of the southern california borderland. *Microb. Ecol.* 53 631–638. 10.1007/s00248-006-9148-3 17345141

[B22] Hoai T. D. Nishiki I. Yoshida T. (2016). Properties and genomic analysis of Lactococcus garvieae lysogenic bacteriophage PLgT-1, a new member of Siphoviridae, with homology to Lactococcus lactis phages. *Virus Res.* 222 13–23. 10.1016/j.virusres.2016.05.021 27234995

[B23] Howard-Varona C. Hargreaves K. R. Abedon S. T. Sullivan M. B. (2017). Lysogeny in nature: mechanisms, impact and ecology of temperate phages. *ISME J.* 11 1511–1520. 10.1038/ismej.2017.16 28291233 PMC5520141

[B24] Hurwitz B. L. U’Ren J. M. (2016). Viral metabolic reprogramming in marine ecosystems. *Curr. Opin. Microbiol.* 31 161–168. 10.1016/j.mib.2016.04.002 27088500

[B25] Iriarte A. Madariaga I. Revilla M. Sarobe A. (2003). Short-term variability in microbial food web dynamics in a shallow tidal estuary. *Aquat. Microb. Ecol.* 31 145–161. 10.3354/ame031145

[B26] James C. E. Davies E. V. Fothergill J. L. Walshaw M. J. Beale C. M. Brockhurst M. A. (2014). Lytic activity by temperate phages of *Pseudomonas aeruginosa* in long-term cystic fibrosis chronic lung infections. *ISME J.* 9 1391–1398. 10.1038/ismej.2014.223 25461970 PMC4351911

[B27] James C. E. Fothergill J. L. Kalwij H. Hall A. J. Cottell J. Brockhurst M. A. (2012). Differential infection properties of three inducible prophages from an epidemic strain of *Pseudomonas aeruginosa*. *BMC Microbiol.* 12:216. 10.1186/1471-2180-12-216 22998633 PMC3544612

[B28] Jiang S. C. Paul J. H. (1996). Occurrence of lysogenic bacteria in marine microbial communities as determined by prophage induction. *Mar. Ecol. Prog. Ser.* 142:27 10.3354/meps142027

[B29] Jiao N. Herndl G. J. Hansell D. A. Benner R. Kattner G. Wilhelm S. W. (2010). Microbial production of recalcitrant dissolved organic matter: long-term carbon storage in the global ocean. *Nat. Rev. Micro.* 8 593–599. 10.1038/nrmicro2386 20601964

[B30] Keshri J. Pradeep Ram A. S. Colombet J. Perriere F. Thouvenot A. Sime-Ngando T. (2017). Differential impact of lytic viruses on the taxonomical resolution of freshwater bacterioplankton community structure. *Water Res.* 124 129–138. 10.1016/j.watres.2017.07.053 28753495

[B31] Kirchman D. L. (2016). Growth rates of microbes in the oceans. *Annu. Rev. Mar. Sci.* 8 285–309. 10.1146/annurev-marine-122414-033938 26195108

[B32] Knowles B. Silveira C. B. Bailey B. A. Barott K. Cantu V. A. Cobián-Güemes A. G. (2016). Lytic to temperate switching of viral communities. *Nature* 531 466–470. 10.1038/nature17193 26982729

[B33] Laber C. P. Hunter J. E. Carvalho F. Collins J. R. Hunter E. J. Schieler B. M. (2018). Coccolithovirus facilitation of carbon export in the North Atlantic. *Nat. Microbiol.* 3 537–547. 10.1038/s41564-018-0128-4 29531367

[B34] Lai J. Y. H. Zhang H. Chiang M. H. Y. Lun C. H. I. Zhang R. Lau S. C. K. (2018). The putative functions of lysogeny in mediating the survivorship of *Escherichia coli* in seawater and marine sediment. *FEMS Microbiol. Ecol.* 94:36. 10.1093/femsec/fix187 29293955

[B35] Liang Y. Li L. Luo T. Zhang Y. Zhang R. Jiao N. (2014). Horizontal and vertical distribution of marine virioplankton: a basin scale investigation based on a global cruise. *PLoS One* 9:e111634. 10.1371/journal.pone.0111634 25365318 PMC4218788

[B36] Liu H. Tan S. Xu J. Guo W. Xia X. Yan Cheung S. (2017). Interactive regulations by viruses and dissolved organic matter on the bacterial community. *Limnol. Oceanogr.* 62 S364–S380. 10.1002/lno.10612

[B37] Liu L. Cai L. Zhang R. (2017). Co-existence of freshwater and marine T4-like myoviruses in a typical subtropical estuary. *FEMS Microbiol. Ecol.* 93:2329. 10.1093/femsec/fix119 29099976

[B38] Luef B. Luef F. Peduzzi P. (2009). Online program “vipcal” for calculating lytic viral production and lysogenic cells based on a viral reduction approach. *Environ. Microbiol. Rep.* 1 78–85. 10.1111/j.1758-2229.2008.00008.x 21151811 PMC2999826

[B39] Malits A. Weinbauer M. G. (2009). Effect of turbulence and viruses on prokaryotic cell size, production and diversity. *Aquat. Microb. Ecol.* 54 243–254. 10.3354/ame01274

[B40] Marie D. Brussaard C. Thyrhaug R. Bratbak G. Vaulot D. (1999). Enumeration of marine viruses in culture and natural samples by flow cytometry. *Appl. Environ. Microbiol.* 65 45–52. 9872758 10.1128/aem.65.1.45-52.1999 PMC90981

[B41] Paul J. H. (2008). Prophages in marine bacteria: dangerous molecular time bombs or the key to survival in the seas? *ISME J.* 2 579–589. 10.1038/ismej.2008.35 18521076

[B42] Payet J. P. Suttle C. A. (2013). To kill or not to kill: the balance between lytic and lysogenic viral infection is driven by trophic status. *Limnol. Oceanogr.* 58 465–474. 10.4319/lo.2013.58.2.0465

[B43] Pradeep Ram A. S. Chaibi-Slouma S. Keshri J. Colombet J. Sime-Ngando T. (2016). Functional responses of bacterioplankton diversity and metabolism to experimental bottom-up and top-down forcings. *Microb. Ecol.* 72 347–358. 10.1007/s00248-016-0782-0 27179523

[B44] Pradeep Ram A. S. Sime-Ngando T. (2008). Functional responses of prokaryotes and viruses to grazer effects and nutrient additions in freshwater microcosms. *ISME J.* 2 498–509. 10.1038/ismej.2008.15 18273065

[B45] Pradeep Ram A. S. Sime-Ngando T. (2010). Resources drive trade-off between viral lifestyles in the plankton: evidence from freshwater microbial microcosms. *Environ. Microbiol.* 12 467–479. 10.1111/j.1462-2920.2009.02088.x 19878265

[B46] Quast C. Pruesse E. Yilmaz P. Gerken J. Schweer T. Yarza P. (2013). The SILVA ribosomal RNA gene database project: improved data processing and web-based tools. *Nucleic Acids Res.* 41 D590–D596. 10.1093/nar/gks1219 23193283 PMC3531112

[B47] Rastelli E. Corinaldesi C. Dell’Anno A. Tangherlini M. Martorelli E. Ingrassia M. (2017). High potential for temperate viruses to drive carbon cycling in chemoautotrophy-dominated shallow-water hydrothermal vents. *Environ. Microbiol.* 19 4432–4446. 10.1111/1462-2920.13890 28805344

[B48] Rastelli E. Corinaldesi C. Petani B. Dell’Anno A. Ciglenečki I. Danovaro R. (2016). Enhanced viral activity and dark CO 2fixation rates under oxygen depletion: the case study of the marine Lake Rogoznica. *Environ. Microbiol.* 18 4511–4522. 10.1111/1462-2920.13484 27501196

[B49] Rowe J. M. Saxton M. A. Cottrell M. T. DeBruyn J. M. Berg G. M. Kirchman D. L. (2008). Constraints on viral production in the Sargasso Sea and North Atlantic. *Aquat. Microb. Ecol.* 52 233–244. 10.3354/ame01231

[B50] Salmond G. P. C. Fineran P. C. (2015). A century of the phage: past, present and future. *Nat. Rev. Micro.* 13 777–786. 10.1038/nrmicro3564 26548913

[B51] Schattenhofer M. Wulf J. Kostadinov I. Glöckner F. O. Zubkov M. V. Fuchs B. M. (2011). Phylogenetic characterisation of picoplanktonic populations with high and low nucleic acid content in the North Atlantic Ocean. *Syst. Appl. Microbiol.* 34 470–475. 10.1016/j.syapm.2011.01.008 21596506

[B52] Schloss P. D. Westcott S. L. Ryabin T. Hall J. R. Hartmann M. Hollister E. B. (2009). Introducing mothur: open-source, platform-independent, community-supported software for describing and comparing microbial communities. *Appl. Environ. Microbiol.* 75 7537–7541. 10.1128/AEM.01541-09 19801464 PMC2786419

[B53] Sheik A. R. Brussaard C. P. D. Lavik G. Lam P. Musat N. Krupke A. (2014). Responses of the coastal bacterial community to viral infection of the algae Phaeocystis globosa. *ISME J.* 8 212–225. 10.1038/ismej.2013.135 23949664 PMC3869014

[B54] Shen D. Jürgens K. Beier S. (2018). Experimental insights into the importance of ecologically dissimilar bacteria to community assembly along a salinity gradient. *Environ. Microbiol.* 20 1170–1184. 10.1111/1462-2920.14059 29393568

[B55] Suttle C. A. (2005). Viruses in the sea. *Nature* 437 356–361. 10.1038/nature04160 16163346

[B56] Suttle C. A. (2007). Marine viruses — major players in the global ecosystem. *Nat. Rev. Micro.* 5 801–812. 10.1038/nrmicro1750 17853907

[B57] Thingstad T. Lignell R. (1997). Theoretical models for the control of bacterial growth rate, abundance, diversity and carbon demand. *Aquat. Microb. Ecol.* 13 19–27. 10.3354/ame013019

[B58] Thingstad T. F. Vage S. Storesund J. E. Sandaa R. A. Giske J. (2014). A theoretical analysis of how strain-specific viruses can control microbial species diversity. *Proc. Natl. Acad. Sci. U. S. A.* 111 7813–7818. 10.1073/pnas.1400909111 24825894 PMC4040589

[B59] Touchon M. Bernheim A. Rocha E. P. (2016). Genetic and life-history traits associated with the distribution of prophages in bacteria. *ISME J.* 10 2744–2754. 10.1038/ismej.2016.47 27015004 PMC5113838

[B60] Vila-Costa M. Gasol J. M. Sharma S. Moran M. A. (2012). Community analysis of high- and low-nucleic acid-containing bacteria in NW Mediterranean coastal waters using 16S rDNA pyrosequencing. *Environ. Microbiol.* 14 1390–1402. 10.1111/j.1462-2920.2012.02720.x 22390635

[B61] Wang X. Kim Y. Ma Q. Hong S. H. Pokusaeva K. Sturino J. M. (2010). Cryptic prophages help bacteria cope with adverse environments. *Nat. Commun.* 1:147. 10.1038/ncomms1146 21266997 PMC3105296

[B62] Wang Y. Liu Y. Wang J. Luo T. Zhang R. Sun J. (2019). Seasonal dynamics of bacterial communities in the surface seawater around subtropical Xiamen Island, China, as determined by 16S rRNA gene profiling. *Mar. Pollut. Bull.* 142 135–144. 10.1016/j.marpolbul.2019.03.035 31232286

[B63] Wei W. Wang N. Cai L. Zhang C. Jiao N. Zhang R. (2019). Impacts of freshwater and seawater mixing on the production and decay of virioplankton in a subtropical estuary. *Microb. Ecol.* 10.1007/s00248-019-01362-2 [Epub ahead of print]. 30972435 PMC6842343

[B64] Weinbauer M. G. (2004). Ecology of prokaryotic viruses. *FEMS Microbiol. Rev.* 28 127–181. 10.1016/j.femsre.2003.08.001 15109783

[B65] Wilhelm S. W. Brigden S. M. Suttle C. A. (2002). A dilution technique for the direct measurement of viral production: a comparison in stratified and tidally mixed coastal waters. *Microb. Ecol.* 43 168–173. 10.1007/s00248-001-1021-9 11984638

[B66] Wilhelm S. W. Suttle C. A. (1999). Viruses and nutrient cycles in the sea. *BioScience* 49:781 10.2307/1313569

[B67] Williamson S. J. McLaughlin M. R. Paul J. H. (2001). Interaction of the HSIC virus with its host: lysogeny or pseudolysogeny? *Appl. Environ. Microbiol.* 67 1682–1688. 10.1128/AEM.67.4.1682-1688.2001 11282621 PMC92785

[B68] Winget D. M. Helton R. R. Williamson K. E. Bench S. R. Williamson S. J. Wommack K. E. (2011). Repeating patterns of virioplankton production within an estuarine ecosystem. *Proc. Natl. Acad. Sci. U. S. A.* 108 11506–11511. 10.1073/pnas.1101907108 21709214 PMC3136265

[B69] Winter C. Bouvier T. Weinbauer M. G. Thingstad T. F. (2010). Trade-Offs between competition and defense specialists among unicellular planktonic organisms: the “killing the winner” hypothesis revisited. *Microbiol. Mol. Biol. Rev.* 74 42–57. 10.1128/MMBR.00034-09 20197498 PMC2832346

[B70] Xu J. Jing H. Kong L. Sun M. Harrison P. J. Liu H. (2013). Effect of seawater–sewage cross-transplants on bacterial metabolism and diversity. *Microb. Ecol.* 66 60–72. 10.1007/s00248-013-0207-2 23494574

[B71] Zhang R. Weinbauer M. G. Qian P.-Y. (2007). Viruses and flagellates sustain apparent richness and reduce biomass accumulation of bacterioplankton in coastal marine waters. *Environ. Microbiol.* 9 3008–3018. 10.1111/j.1462-2920.2007.01410.x 17991029

[B72] Zheng Q. Wang Y. Xie R. Lang A. S. Liu Y. Lu J. (2018). Dynamics of heterotrophic bacterial assemblages within synechococcus cultures. *Appl. Environ. Microbiol.* 84 e1517–e1517. 10.1128/AEM.01517-1517 29150500 PMC5772231

