# Supplementary material for: Viral Regulation on Bacterial Community Impacted by Lysis-Lysogeny Switch: A Microcosm Experiment in Eutrophic Coastal Waters
Source: Front Microbiol. 2019 Jul 31;10:1763. doi: 10.3389/fmicb.2019.01763 (PMC6685395; doi:10.3389/fmicb.2019.01763)
Supplement: Supplementary file 1 [file Data_Sheet_1.PDF]

Supplementary Information

Viral Regulation on Bacterial Community Impacted by Lysis-Lysogeny Switch: A Microcosm Experiment in Eutrophic Coastal Waters

Xiaowei Chen[1], Ruijie Ma[1], Yunlan Yang[1,2], Nianzhi Jiao[1,*], Rui Zhang[1,*]

[1]State Key Laboratory of Marine Environmental Science, College of Ocean and Earth Sciences, Institute of Marine Microbes and Ecospheres, Xiamen University, Xiamen 361102, PR China

[2]College of the Environment and Ecology, Xiamen University, Xiamen 361102, PR China

***Corresponding authors:**

Rui Zhang (Email: ruizhang@xmu.edu.cn)

Nianzhi Jiao (Email: jiao@xmu.edu.cn)

This supplementary information includes 5 pages, 3 figures and 1 table.

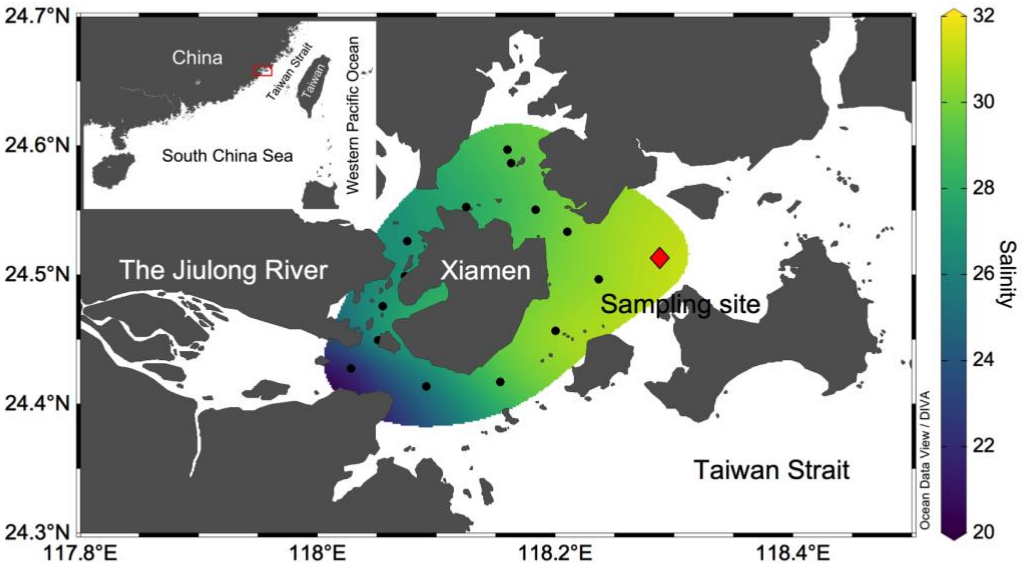

Supplementary Figure S1. Sampling site (red diamond) in Xiamen Bay. The base map is colored according to the annual mean water salinity values of 15 long-term monthly sampling stations (black dots) in 2015. The map was generated using Ocean Data View (version 5.1.7, Schlitzer, R., 2019).

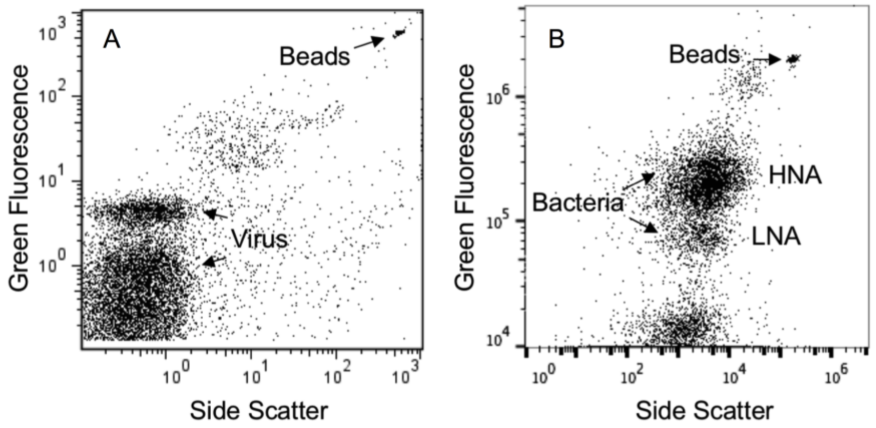

Supplementary Figure S2. Cytometric plot graph of the viral (A) and bacterial (B) populations in samples from Xiamen Bay. The total bacterial population contains cells with high nucleic acid (HNA) and low nucleic acid (LNA) contents.

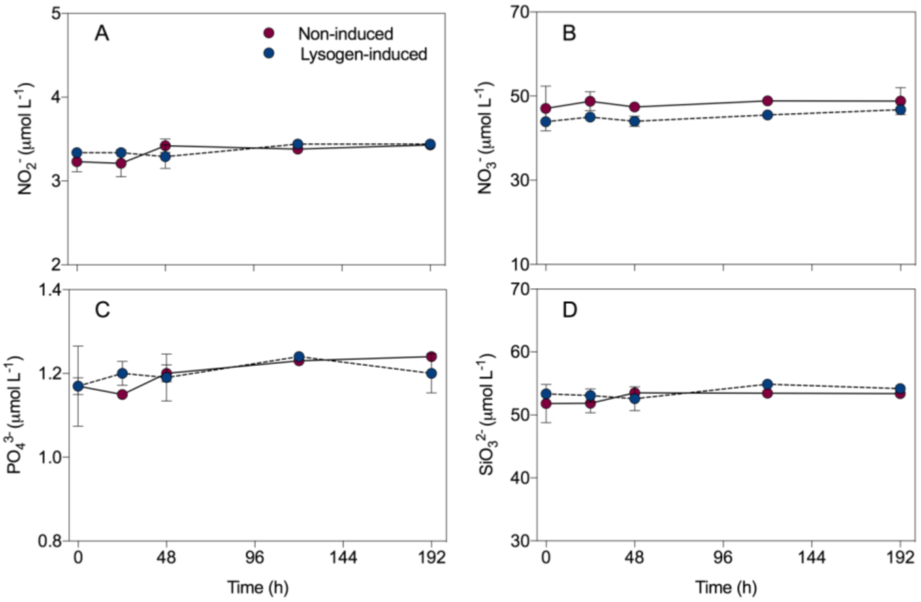

Supplementary Figure S3. Dynamics overtime of NO_2^- (A), NO_3^- (B), PO_4^{3-} (C) and SiO_3^{2-} (D) in the non-induced and lysogen-induced treatments. The error bars are indicated as standard deviation (SD).

Supplementary Table S1. Regression relationships among the log-transformed viral abundance, bacterial abundance, the abundances of high nucleic acid (HNA) and low nucleic acid (LNA) cells, lytic and lysogenic viral production.

Treatment	Dependent Variable	Independent Variable	Intersect	Slope	R^2	*P*
Non-induced	Bacterial abundance	Viral abundance	6.650	-0.019	0.001	0.893
Lysogen-induced	Bacterial abundance	Viral abundance	7.181	-0.117	0.134	0.149
Non-induced	HNA abundance	Viral abundance	7.366	-0.144	0.309	**0.021***
Lysogen-induced	HNA abundance	Viral abundance	6.806	-0.06	0.467	**0.003****
Non-induced	LNA abundance	Viral abundance	3.948	0.427	0.572	**<0.001****
Lysogen-induced	LNA abundance	Viral abundance	5.964	0.085	0.051	0.381
Non-induced	Bacterial abundance	Lytic VP	3038	0.237	0.032	0.772
Non-induced	Bacterial abundance	Lysogenic VP	-1.28	0.932	0.991	**<0.001****
Lysogen-induced	Bacterial abundance	Lytic VP	4.691	-0.052	0.005	0.904
Lysogen-induced	Bacterial abundance	Lysogenic VP	7.847	-0.570	0.542	0.156
Non-induced	HNA abundance	Lytic VP	5.802	-0.224	0.1146	0.577
Non-induced	HNA abundance	Lysogenic VP	2.325	0.389	0.6857	0.083
Lysogen-induced	HNA abundance	Lytic VP	4.881	-0.094	0.212	0.435
Lysogen-induced	HNA abundance	Lysogenic VP	5.416	-0.191	0.688	0.082
Non-induced	LNA abundance	Lytic VP	-4.110	1.425	0.794	**0.042***
Non-induced	LNA abundance	Lysogenic VP	0.909	0.602	0.280	0.359
Lysogen-induced	LNA abundance	Lytic VP	1.798	0.435	0.241	0.401
Lysogen-induced	LNA abundance	Lysogenic VP	6.020	-0.274	0.076	0.653

VP for viral production and the bold number mean it is significant. *$P \leq 0.05$; **$P \leq 0.01$.
